# Video-assisted thoracoscopic surgery simulation and training: a comprehensive literature review

**DOI:** 10.1186/s12909-023-04482-z

**Published:** 2023-07-27

**Authors:** Sarah Grossi, Maria Cattoni, Nicola Rotolo, Andrea Imperatori

**Affiliations:** 1 Center for Thoracic Surgery, Department of Medicine and Surgery, University of Insubria, Via Guicciardini, 9, Varese, 21100 Italy; 2 Center for Minimally Invasive Surgery, Department of Medicine and Surgery, University of Insubria, Varese, Italy

**Keywords:** Video-assisted thoracic surgery, VATS, Simulators, Training, Curriculum.

## Abstract

**Background:**

Video-assisted thoracic surgery (VATS) has become the standard for lung cancer diagnosis and treatment. However, this surgical technique requires specific and dedicated training. In the past 20 years, several simulator systems have been developed to promote VATS training. Advances in virtual reality may facilitate its integration into the VATS training curriculum. The present review aims to first provide a comprehensive overview of the simulators for thoracoscopic surgery, focused especially on simulators for lung lobectomy; second, it explores the role and highlights the possible efficacy of these simulators in the surgical trainee curriculum.

**Methods:**

A literature search was conducted in the PubMed, EMBASE, Science Direct, Scopus and Web of Science databases using the following keywords combined with Boolean operators “AND” and “OR”: virtual reality, VR, augmented reality, virtual simulation, mixed reality, extended reality, thoracic surgery, thoracoscopy, VATS, video-assisted thoracoscopic surgery, simulation, simulator, simulators, training, and education. Reference lists of the identified articles were hand-searched for additional relevant articles to be included in this review.

**Results:**

Different types of simulators have been used for VATS training: synthetic lung models (dry simulators); live animals or animal tissues (wet simulators); and simulators based on virtual or augmented reality. Their role in surgical training has been generally defined as useful. However, not enough data are available to ascertain which type is the most appropriate.

**Conclusions:**

Simulator application in the field of medical education could revolutionize the regular surgical training curriculum. Further studies are required to better define their impact on surgeons’ training programs and, finally, on patients’ quality of care.

**Supplementary Information:**

The online version contains supplementary material available at 10.1186/s12909-023-04482-z.

## Background

Since the first video-assisted thoracic surgery (VATS) lung lobectomy was performed in 1991 [1], the use of VATS has significantly increased, and it is now the gold standard for diagnosis and treatment in thoracic surgery [2–4].

Compared to thoracotomy, the VATS approach, by single or multiple ports, has been demonstrated to have advantages, including less postoperative pain, fewer complications, enhanced postoperative recovery, shorter hospitalisation, better tolerance to adjuvant chemotherapy and better quality of life [5–10].

However, this technique requires the use of a camera to view the surgical field and is more complex than the standard thoracotomy approach, with a potential major risk of damaging vessels or other vital structures with fatal consequences for the patient [11]. Learning this technique requires continuous and specific training: approximately 50 procedures performed in 1 year are required to overcome the learning curve. Currently, most education takes place directly on patients during daily surgical activity under the responsibility of a senior surgeon; hands-on training is infrequent [12, 13]. Thus, different types of simulators have been developed and proposed over the years to facilitate the acquisition of the skills required to perform the VATS lobectomy technique in a risk-free environment for trainees and especially patients.

The present review aims to provide a comprehensive overview of the different simulators proposed for VATS lobectomy training. Second, it explores the role and highlights the possible efficacy of these simulators in thoracic surgical training.

### Methods

The literature search was performed according to the PRISMA guidelines [14] and based on the PubMed, EMBASE, Science Direct, Scopus and Web of Science databases; the following keywords combined with Boolean operators “AND” and “OR” were used: virtual reality, VR, augmented reality, virtual simulation, mixed reality, extended reality, thoracic surgery, thoracoscopy, VATS, video-assisted thoracoscopic surgery, simulation, simulator, simulators, training, and education (see Appendix). No year of publication limit was set. Only English texts and original articles were included.

After the removal of duplicates, a review of titles and abstracts was conducted to identify articles of potential interest. These were retrieved for full-text analysis and included if deemed relevant. Reference lists were hand-searched for additional relevant studies to include in this review. Studies focused on other surgeries, open thoracic surgery or paediatric surgery were excluded; thus, only articles focused on simulators for VATS training were included.

The heterogeneity of the included studies prompted us to synthesize the obtained data narratively.

This review did not involve human subject research, so institutional review board approval was not needed.

## Results

**Fig. 1:**
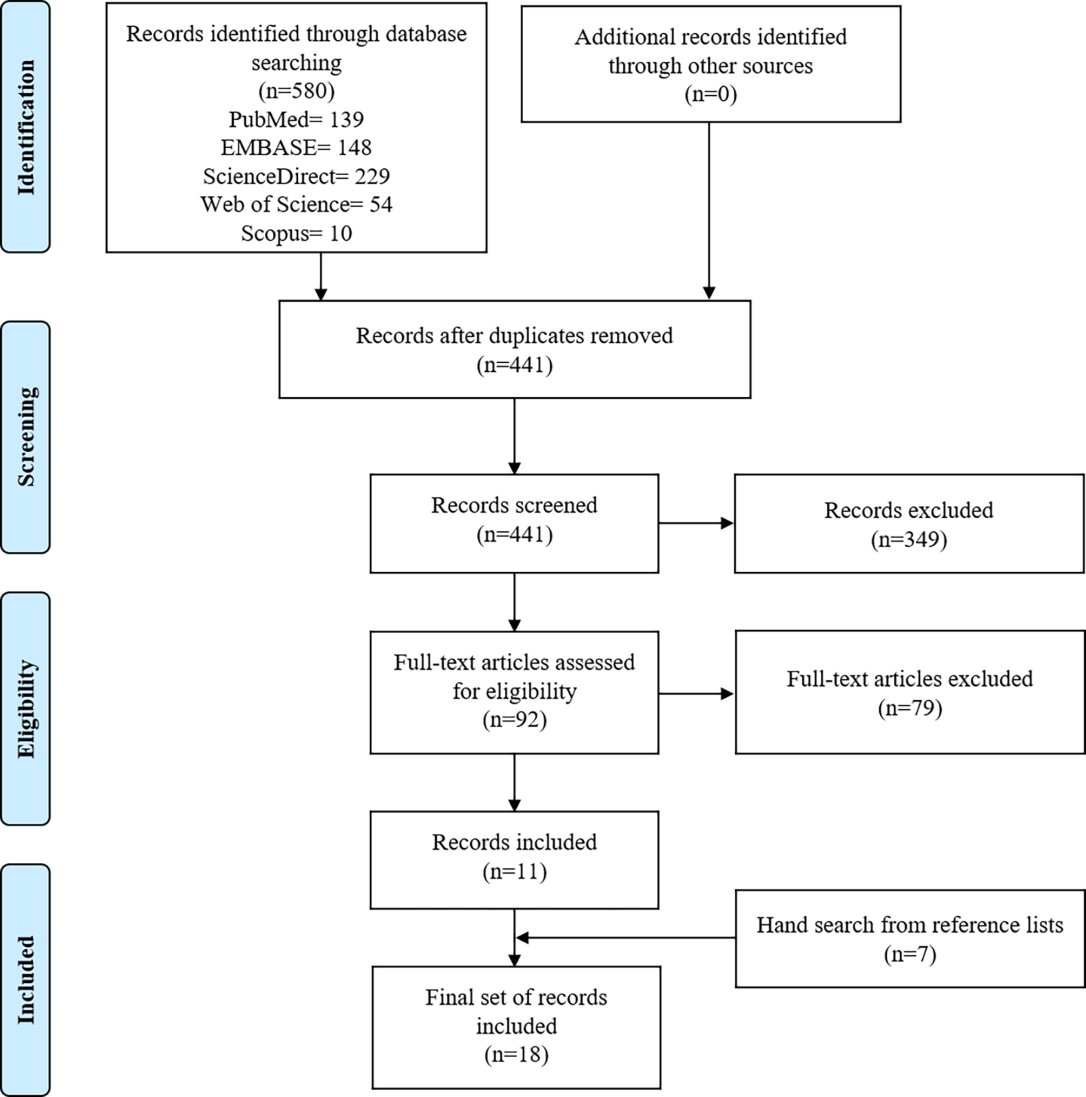
PRISMA flow chart of included studies

The initial database search revealed 580 records published between August 1978 and February 2022 (last update: February 18, 2022). The study inclusion process is summarised in Fig. 1.

Eighteen original articles were defined as eligible for inclusion in this review [15–32]. The main characteristics of the studies are summarised in Table 1.

**Table 1:** Included studies main characteristics

Ref.	Participants (n)	Simulator device	Assessed Tasks	Outcomes
15	12 surgeons	Human cadaver	VATS lobectomy (3-port anterior approach)	Time, questionnaire
16		Anaesthetized swine	VATS upper left lobectomy (3-port anterior approach)	Time, hypoventilation and bradycardia rate, mortality
17		Sheep	VATS lobectomy (uniportal approach)	
18	40 (chief physicians, senior doctors, consultants and residents)	Trainer box: Tuebingen Thorax Trainer (TuThor)	Preparation of the hilum structures, bronchia and lymphadenectomy; ligation, clipping and use of the MicroCutter device; segment resection; suturing and anastomosis of the bronchia and free hands-on training	Questionnaire on “The used training setup is suitable for video-assisted thoracic surgery training” opinions
19	> 100 participants with previous clinical experience in thoracoscopic surgery and 17 faculty members	Training box: porcine heart-lung tissue blocks	VATS lobectomy	Qualitative data collected from faculty and course participants on simulator opinions
20	31 (novices = 13, intermediates = 6, experts = 12)	Training box: porcine heart-lung tissue blocks	VATS left upper lobectomy	Time, errors, Likert scale and questionnaire on simulator evaluation in terms of fidelity and content validity
21	13 (senior cardiothoracic surgeons)	Training box: a left porcine heart–lung block placed within the chest cavity of a mannequin	VATS left lobectomy	Questionnaire and rating scales for performance assessment based on the OSATS
22	55 (surgeons = 50; chest specialists = 5)	Training box: consisted of a blood flow source, vessels, bronchus, lung, and a human hemibody all made with synthetic materials	VATS right upper lobectomy	/
23		Training box: consisted of a polyvinyl-alcohol (PVA) hydrogel mimicking real human anatomy and texture	VATS right lower lobe lobectomy, right upper lobe lobectomy and mediastinal lymph node dissection	/
24	40 (Surgeons = 10; Control group = 10; self-guided group = 10; educator-guided group = 10)	Training box: D-Box Basic Simulator (SimSurgery AS, Norway)	3 task and then removing an artificial tumor from a porcine lung by wedge resection	Time, modified version of OSATS
25		VR simulator: gaming laptop PC + haptic feedback device used to control the surgical instruments	VATS right upper lobe resection	/
26	28 (residents)	Trainer box: D-BOX basic simulator. VR simulator: SEP (SimSurgery)	Remotion of a left upper lobe on a porcine heart and lung block on D-BOX simulator	Performance scores based on time and errors
27	103 (novices = 32, intermediates = 45, experts = 26)	VR simulator: LapSim (Surgical Science, Gothenburg, Sweden),	VATS lobectomy of the right upper lobe using a three-port standardized anterior approach	Questionnaire on content validity, time, blood loss, right and left instrument pathway, right and left instrument cumulated degrees
28	53 (novices = 17,intermediates = 22, experts = 14)	VR simulator: LapSim (Surgical Science, Gothenburg, Sweden)	VATS right upper lobectomy (3 port anterior approach)	Time, blood loss, right/left instrument pathway length /angle, number of tool switches, stretch damage on vessel/ bronchus to middle/superior lobe, stretch damage on middle/superior lobe, number of vessels severed, number of time stapled on lobes, number of structures stapled without removing yellow band, number of time bronchus is crushed before stapling, number of incorrect stable reload used for bronchus/vessels
29	41 (novices = 22, intermediates = 10, experts = 9)	VR simulator: LapSim (Surgical Science, Gothenburg, Sweden)	VATS lobectomy for three different randomly chosen lobes utilizing the Copenhagen Standardized Anterior Approach.	Time, blood loss right/left/total instrument path length, right/left/total instrument angle movements, stretch damage on vessel/bronchus/lobe
30	20 (trainees = 12, consultant = 8)	VR simulator: Lap Mentor simulator (Simbionix Products, Surgical Science, Gothenburg, Sweden)	VATS right upper lobectomy	OSATS, GOATS, NASA-TLX, and a comprehensive evaluation questionnaire
31	113 (surgeons in training = 85, medical students = 28)	VR simulator: single hole board for single incisional laparoscopic surgery (Covidien, USA) + a surgical video system (Viking Systems, Inc., USA)	Peg transfer, needle transfer, suturing	Time and failure rate
32	30 (novices = 24, experts = 6)	VATS-AR simulator	Peg transfer, vascular clipping and shearing, and rope perforation	Total operation time, clamps track length, numbers of block drop, the five-point Likert scale for simulator performance evaluation

Abbreviations: video-assisted thoracic surgery (VATS); virtual reality (VR); Objective Structured Assessment of Technical Skill (OSATS); Global Operative Assessment of Thoracoscopic Skills (GOATS); National Aeronautics Space Administration-Task Load Index (NASA-TLX).

Simulators are classified into three groups according to the system used to recreate the human chest and its contents: wet simulators (animal tissues or live animals), dry simulators (synthetic lung models), and those requiring virtual reality (VR) or augmented reality (AR).

### Human cadavers and live anaesthetised animals

Dell’Amore and collaborators reported their experience with the use of human cadavers to simulate three-portal VATS lobectomy [15]. In their study, the cannulation technique interestingly allowed the blood vessels to fill in a realistic way, demonstrating that this model could be effective for VATS training. The authors concluded that it could be considered a good alternative to live anaesthetized animal models because all those who have trained on this model were able to complete all step of the operation and the score obtained from the questionnaire about the quality of the VATS simulation performed was high (the median total score was 40.5 out of a maximum score of 50) [15].

As an alternative to human cadavers, which often have limited availability, swine are one of the most commonly used animal models for surgical training, given their anatomical and physiological similarities with humans. Tedde et al. described the results of VATS lobectomy performed on 40 live swine, underlying the anatomical differences and the complications due to anaesthesia and to single lung ventilation: 26 animals (65%) developed intraoperative hypoventilation, and 4 (10%) of them died before the end of surgery; 8 (20%) had bradycardia, with 2 (5%) deaths due to this complication [16].

Sheep have also been introduced as an animal model for VATS lobectomy training; according to users, sheep present an anatomy more similar to humans than swine. Following their experience with different courses in The Technological Center (Coruña University Hospital, Spain), de la Torre et al. affirmed that sheep was an excellent animal model [17].

During surgical simulations on live animals, the presence of a veterinarian and an anaesthetist is required [16, 17]. It has also been reported that the use of live animals for surgical training is not unequivocally accepted. Indeed, there are groups of animal rights activists, mainly in the United States and Europe, who fight against the use of animals for experiments and surgical training [33]. Among the large-scale lobbying activities organised by these groups, an example is the “Stop Vivi-Section” initiative of 2015 [33, 34].

### Wet and dry trainer boxes

Among the trainer boxes, Domhan and collaborators developed TuThor, based on a complete porcine heart-lung complex where the swine anatomic details have been combined with a perfusion system and a rotatable thoracic cage based on human anatomy. This training device highly resembles the thoracic surgical field. Indeed, it was tested at the four hands-on training courses (Tübingen University Hospital, Germany) by 40 participants, who stated that it was a suitable model for VATS training (87.5%) and that it was realistic for the level of detail and scale (76%). Moreover, it has low production cost and no ethical concerns since the tissues used are a product of meat processing [18].

Meyerson et al. [19] proposed a trainer box based on a porcine heart-lung tissue block designed with the left lung up in a plastic box with multiple holes simulating the different combinations of VATS incisions. Pulmonary arteries and veins are individually suffused with ketchup to simulate bleeding. This simulator was tested by 100 participants and 17 faculty members who provided qualitative feedback: this model was found inexpensive, easy to produce and effective for training surgeons at any level. Moreover, this simulator was tested by 31 residents (12 experienced, 6 intermediate and 13 novice) and shows acceptable fidelity, content, and construct validity. In addition, the 12 experienced participants were able to successfully complete the lobectomy, whereas only 4 of 6 intermediate and 5 of 13 novices completed the surgical procedure, so this simulator demonstrated to be able to distinguish between the competency of its users [20]. Thanks to these positive characteristics, in 2016, this trainer box was introduced in the Thoracic Surgery Department of Salamanca as part of a training program for minimally invasive surgery [35, 36].

Fann and colleagues developed 12 simulators for cardiac or thoracic surgical simulation. In detail, for VATS lobectomy, they used a left porcine heart-lung block placed in the chest cavity of a mannequin with fixed working ports [21]. This simulator allows the identification of anatomic landmarks, manoeuvring of the thoracoscope and pulmonary structures, dissection and encircling of hilar vessels and bronchus and division of the structures using endoscopic staplers. According to the study participants, this simulator was more complex than a clinical case due to the interspecies differences, but it allowed the simulation of many advanced manoeuvres [21].

Iwasaki’s group was one of the first to develop a model to assist training in VATS right upper lobectomy using only polyvinyl chloride components with pulsatile lungs in the absence of anaesthetized live animals. Fifty thoracic surgeons and five chest specialists who tested this simulator experienced tension resulting from injury to vessels and proved the pulsation of artificial blood flow. Indeed, the main components of this model were a blood flow source, vessels, bronchus, lung, and a human hemi-body; the mechanism of this training simulator was based on vessels with circulating blood in a lung covered with a plastic replica of the human body [22].

More recently, Morikawa and collaborators described their three-dimensional rib cage and polyvinyl-alcohol hydrogel lung model, with a silicon-based “skin” flap that covers the model and in which the trainee can perform incisions for ports or access windows [23, 37]. This model could be considered an evolution of the model described in Iwasaki’s work [22], with a more realistic but more expensive structure and texture [37].

Finally, Bjurstrom and collaborators investigated the effect of training with the D-Box Basic Simulator (SimSurgery AS, Norway), a commercial video-trainer simulator, based on three scenarios of increasing fidelity and difficulty, with and without a dedicated educator. Using a modified version of a validated assessment tool, two thoracoscopic experts blindly and independently recorded and assessed the final standardised test (VATS lung wedge resection). Intensive simulator training with a dedicated educator has been shown to enable novices to perform an acceptable wedge resection in a simulation model. Moreover, although the difference observed in the final score was not significant, it should be underlined that the presence of the educator during the training led to a positive effect [24].

### Introduction of virtual and augmented reality in the simulator systems

Regarding the VR-based simulators developed for training in VATS lobectomy, the first to be described was the one developed by Solomon and collaborators [25]. It included a standard laptop computer and a haptic feedback device used to control the surgical instruments. This simulator allowed different anatomic variations and anomalies to be loaded, and the software was designed to identify common errors. However, this simulator was a hybrid between low- and high-fidelity models, and its commercial distribution required two years [25].

Since no VR simulators were commercially available for VATS training in early 2010, Jensen and collaborators compared the SimSurgery VR simulator to the box trainer to investigate whether training on a simulator for laparoscopic surgery allowed trainees to perform thoracoscopic lobectomy [26]. The results showed that training for a nephrectomy (task chosen because it was similar to the thoracoscopic lobectomy that was not included in the VR simulator software) on a laparoscopic VR simulator added no advantage over box training. Moreover, the skills learned on the laparoscopy VR simulator were less transportable to the heart-lung block than those learned on the trainer box simulator [26].

Thus, in collaboration with Surgical Science Specialists, Jensen and collaborators developed VATS lobectomy software for the LapSim VR simulator (Surgical Science, Gothenburg, Sweden). This simulator was presented and tested at the 22nd meeting of the European Society of Thoracic Surgeons (ESTS – Copenhagen, Denmark, 2014), where it was found to be realistic and to have good content validity [27]. After a revision of the software, its validity in simulating VATS lobectomy was assessed [28]. Then, it was tested by novices and experienced surgeons, who found that the angulation of the right instrument and 10/19 built-in simulator metrics (including time, instrument path length, damage to vessels and errors) were significantly different between novices and experienced surgeons. These variables were chosen to establish a pass/fail level that could be used to assess thoracic surgery trainees’ VATS lobectomy competency [28].

More recently, Haidari and collaborators investigated the validity evidence for the new VATS lobectomy modules for the LapSim simulator with haptic feedback (Surgical Science, Gothenburg, Sweden), including all five lobectomies [29]. Forty-one participants (novices n = 22, intermediates n = 10, experts n = 9) performed three consecutive simulated VATS lobectomies of randomly selected lobes, for a total of 123 lobectomies. In this study, 3 metrics (time, blood loss, and total instrument path length) showed a significant difference between experienced surgeons and novices, supporting their use in assessing VATS lobectomy competency for trainees in thoracic surgery.

Another simulator system assessed for thoracic surgery training was the Lap Mentor simulator (Simbionix Products, Surgical Science, Gothenburg, Sweden), which includes the lobectomy module for VATS right upper lobectomy using the anterior approach. This simulator was used to create a VR curriculum representing an evidence-based approach for VATS training programs by Bedetti and collaborators [30]. Basic skills were tested using the Objective Structured Assessment of Technical Skill (OSATS) and the Global Operative Assessment of Thoracoscopic Skills (GOATS). The surgical performance of twenty volunteers divided into trainees (n = 12) and consultants (n = 8) was assessed and compared, but no significant difference was observed between the two groups. However, this study supported the inclusion of VR simulation in surgical training programs, underlining that the experience gained in the operating theatre cannot be replaced by VR simulation.

Finally, Han and collaborators investigated the effectiveness of 3D displays in uniportal VATS training, hypothesising that the improved depth perception provided by 3D displays might be emphasised in the uniportal approach. A total of 113 trainees (85 surgeons in training and 28 medical students) completed three basic surgical skills under 2D and 3D video systems [31]. The simulation system consisted of a 3-cm single-hole board used for laparoscopy training (Covidien, Norwalk, CT, USA), the training module and the endoscopic devices. The results showed that the 3D video system reduced the time performance and the number of errors compared to the 2D system. Participants indicated that the 3D display was advantageous due to the better depth perception and the consequent better endoscopic device handling.

To conclude, an AR-based visual haptic modelling system was recently developed for VATS training. The tactile and visual senses, authenticity and simulator performance were assessed by using face, content, and construct validation methods. The simulator was demonstrated to be useful as a training device to assist novices in thoracoscopic skills development [32].

## Discussion

Currently, VATS is the basic standard of care for lung disease, especially for surgical treatment of early-stage lung cancer. However, the adoption of this technique is challenging because of the fulcrum effect, the loss of direct tactile sensation and the need to convert two-dimensional images into three-dimensional perception. Moreover, the potential risk of intraoperative haemorrhage requires adequate management skills to avoid further complications [11, 38]. To minimise this risk, trainees should reach a predefined level of proficiency in VATS before operating on patients [29].

Even qualified surgeons may have difficulty in learning this technique, as reported by Ra et al. [39]. Indeed, despite the experience gained after performing 100 open lobectomies, the surgeon under review showed statistically significant improvements in his learning curve after six months. On the other hand, about the analysis of the learning curves of trainee physicians, it was reported that only after performing 50 open lung resections they were able to achieve an average operative time similar to that of their consultant [40].

Thus, to reduce the learning curve time, thoracic surgery educators are currently looking towards useful educational models, including the use of clinical simulators, to improve cognitive and procedural skills before trainees operate on patients [25].

The human cadaver appears to be the most realistic model due to its anatomical correspondence. It allows simulation of patient positioning and trocar placement, but the quality of tissues is poor. Moreover, the absence of vascular distension and the poor preservation of the cadaver could result in difficulties in identifying the operative landmarks [16, 18].

In general, animals are the most commonly used model for surgical training when a new surgical technique is developed [41]. The swine animal model is most frequently used to simulate VATS lobectomy and surgical procedures in general [16]. This model allows realistic tissue handling and the possibility of simulating critical conditions, such as bleeding, in a risk-free clinical scenario [19]. However, there are main differences between swine and human anatomy. The porcine thoracic cavity is laterally compressed, cone-shaped, and not dorsoventrally compressed as in humans. Moreover, in the swine lung, the right cranial lobe bronchus originates directly from the trachea before its bifurcation, and there are no hilar or mediastinal lymph nodes [19, 42, 43].

Sheep are also an excellent model for VATS lobectomy training, despite the following anatomical differences: the thoracic cavity is wider than the human thoracic cavity, the left upper lobe is smaller, and the lingula is longer. On the right side, there is a large cava vein with the pulmonary artery and both pulmonary veins hidden behind it [16, 17]. Although sheep have more human-like anatomy than swine, they are rarely used as animal models for surgical training due to their high cost.

Although live animals perform well in thoracic surgery training, their use implies some problems, first, the need for ethics committee authorisation, and in some countries, their use is prohibited [44]. Moreover, the revised Directive 2010/63/EU for the protection of laboratory animals has officially implemented the 3R principles (reduction, refinement, replacement) into European law [45], highlighting the necessity to replace animals with other training systems despite the belief that the use of live animals is crucial for correct surgical training and that adequate alternative models have not yet been developed [46]. This need for more attention to animal welfare and accordance with the 3R principles has led to research on alternative methods to substitute live animals with other training models, such as surgical simulators.

The trainer box, based on artificial or ex vivo organs, has been proven to be quite realistic and easy to use [19, 35, 47]. Trainer boxes based on porcine heart-lung tissue blocks have been demonstrated to be slightly inferior to live porcine models but better than cadaver models in terms of tissue quality and vessel management [19]. In general, training box simulators are inexpensive and require only a few instruments, a monitor and a scenario of some kind, such as a swine lung, to be placed in the training box [19, 48]. However, most of the training boxes cannot simulate bleeding complications because they lack organ perfusion. This is an important deficiency because in VATS, the capability of haemorrhage control is fundamental since it is not a rare complication [18].

Conversely, VR simulators can simulate bleeding or anatomical variations and are ready to use, but they are more expensive [26, 30]. Important advantages of VR simulators are automated feedback and instruction modules, but the haptic force of a VR simulator is less realistic since it is mechanically simulated. Thus, the trainer box is superior to the VR simulator in terms of haptic feedback, but it needs a dedicated instructor to record trainees’ measurements and to let trainees take advantage of simulator benefits [24, 27].

Finally, simulators based on AR have proven to be very useful for surgical training [32]. Compared to the VR simulator, the AR system has more realistic surgical training environments, the visuohaptic experience is closer to that of human factors engineering, and the immersive interactive perception appears more natural [49].

It is also important to mention the presence of high heterogeneity in techniques and quantification of results reported in the studies mentioned in this paper. Indeed, there is still disagreement about the central steps of the procedure or the best ways to teach VATS. For this reason, recent studies have used the Delphi process in order to identify the essential steps of VATS lobectomy, the main difficulties encountered in their execution, and finally the most appropriate areas to focus on during the simulation phase [50, 51].

For these reasons, the attention of thoracic surgery societies is focusing on the development of tests that accurately assess surgical competence for VATS and particularly on the development and validation of a new VATS lobectomy assessment tool, which could become an important aid in the training and certification of future thoracic surgeons [28, 29, 38, 52, 53].

Finally, it is important to emphasize that in addition to training in surgical skills, non-technical skills (NTS), including planning and preparation, situation awareness, and leadership, were also crucial for technique performance.

Regarding VATS lobectomy, Gjeraa et al. identified six NTS that were perceived as important during this surgical procedure (planning and preparation, situation awareness, problem solving, leadership, risk assessment, and teamwork). Authors concluded that, despite these NTS not being considered essential for a safe and successful procedure, they should be considered important because they contribute to the team’s Shared Mental Models in relation to the patient, the current situation, and team resources [54]. Subsequently, the same group analysed fifty-eight lobectomy procedures and highlighted how a better team’s Shared Mental Models was related to a significantly shorter duration of surgery and decreased intraoperative bleeding [55].

However, no dedicated thoracic surgery simulation program has been designed to teach vital skills.

Despite the different nature of the available simulators, all the authors agree that simulators play an important role in VATS training, but which type of simulator is the most instructive is still a matter of debate.

## Conclusions

Simulator-based learning has enormous potential to revolutionize surgical training. It is necessary to emphasise that real-life clinical experience cannot be completely replaced by any simulator. Thus, the simulator could be considered a sort of integrative tool to train novices in everyday surgical activities and to accelerate their VATS learning curve.

Although the potential application of simulators in the field of medical education is notable, further efforts are required to assess their effective contribution to surgeons’ training and to patients’ quality of care and safety. Finally, more data are necessary to identify the ideal simulator model, compare outcomes during and after residents’ learning curve, and propose an efficient simulation training program with validated measures of trainees’ performance.

### Electronic supplementary material

Below is the link to the electronic supplementary material.

Supplementary Material 1

## Data Availability

All data generated or analysed during this study are included in this published article.

## List of abbreviations

VATS: Video-assisted thoracic surgery
VR: Virtual Reality
AR: Augmented Reality
ESTS: European Society of Thoracic Surgeons
OSATS: Objective Structured Assessment of Technical Skill
GOATS: Global Operative Assessment of Thoracoscopic Skills
NTS: Non-technical skills
