# Supplementary material for: Video-assisted thoracoscopic surgery simulation and training: a comprehensive literature review
Source: BMC Med Educ. 2023 Jul 27;23:535. doi: 10.1186/s12909-023-04482-z (PMC10375656; doi:10.1186/s12909-023-04482-z)
Supplement: Supplementary file 1 — Supplementary Material 1 [file 12909_2023_4482_MOESM1_ESM.docx]

**Example search strategy used to retrieve relevant journal articles**

PubMed = 139

((thoracic surgery OR thoracoscopy OR VATS OR Video assisted thoracoscopic surgery) AND (training OR education) AND (Virtual reality OR augmented reality OR virtual simulation OR mixed reality OR extended reality OR VR) AND (simulation OR simulator OR simulators))

EMBASE = 148

((thoracic surgery OR thoracoscopy OR VATS OR Video assisted thoracoscopic surgery) AND (training OR education) AND (Virtual reality OR augmented reality OR virtual simulation OR mixed reality OR extended reality OR VR) AND (simulation OR simulator OR simulators))

ScienceDirect = 229

(thoracic surgery OR minimal invasive surgery OR thoracoscopy OR VATS) AND (virtual reality OR VR) AND (simulator) AND (training)

Scopus = 10

( ( ( thoracic AND surgery OR thoracoscopy OR vats OR video AND assisted AND thoracoscopic AND surgery ) AND ( virtual AND reality OR augmented AND reality OR virtual AND simulation OR mixed AND reality OR extended AND reality OR var ) AND ( simulation OR simulator OR simulators ) AND ( training OR education ) ) )

Web Of Science = 54

((thoracic surgery OR thoracoscopy OR VATS OR Video assisted thoracoscopic surgery) AND (training OR education) AND (Virtual reality OR augmented reality OR virtual simulation OR mixed reality OR extended reality OR VR) AND (simulation OR simulator OR simulators))

Only English texts and original articles were included
